# Cyclic stroke mortality variations follow sunspot patterns

**DOI:** 10.12688/f1000research.24794.2

**Published:** 2020-12-22

**Authors:** Stella Geronikolou, Alexandros Leontitsis, Vasilis Petropoulos, Constantinos Davos, Dennis Cokkinos, George Chrousos

**Affiliations:** 1 Clinical, Translational and Experimental Surgery, Biomedical Research Foundation of the Academy of Athens, Athens, 11527, Greece; 2 University of Ioannina, Ioannina, Greece; 3 Research Center for Astronomy and Applied Mathematics, Academy of Athens, Athens, 11527, Greece; 4 University Research Institute of Maternal and Child Health and Precision Medicine, UNESCO Chair on Adolescent Health Care, National and Kapodistrian University of Athens, Thivon and Levadeias, Athens, 11527, Greece

**Keywords:** Sunspot numbers, Chronome, Stroke mortality, Singular Spectrum Approach, NCOR1, R1 interactome

## Abstract

Background: Mapping time-structures is a burgeoning scientific field enriching the (P4) medicine models. Local evidence in Mediterranean populations is underinvestigated. Methods: The Censused stroke-related death events (D) in the largest East-Mediterranean port (Piraeus), during (1985-1989), when local population had diet (low fat/sugar, proteins and vegetables/fruits daily, and pure olive oil almost exclusively) and genetic homogeneity-later interrupted by the immigration into Greece in 1990; and Sunspot numbers were indexed by Wolf numbers (Rz) (1944-2004), and evaluated using Fast Fourier Analysis and Singular Spectrum Analysis in MATLAB. Results: D were turned with fluctuations >35% in Rz. A non-anthropogenic 6.8 days cycle was recognized. Conclusions: This study may be taken into consideration in future public health planning and chronotherapy evaluations.

## Glossary

aa index: a simple global geomagnetic activity index derived from the *K* indices from two approximately antipodal observatories and has units of 1 nT.

Census: The total count of a given population (its characteristics included) on a certain date.

Census (verb): to take a census of a population in a city, region, country etc.

K _p_ index: The global geomagnetic activity index that is based on 3-hour measurements from ground-based magnetometers around the world. It characterizes the magnitude of geomagnetic storms.

P4 medicine: predictive, preventative, personalized, and participatory medicine- a proactive model of medicine, coined by Hood and Friend in 2011.

Smoothed (line in a plot): the data points of a signal modified so that individual points higher than the adjacent points (presumably because of noise) are reduced, while points that are lower than the adjacent points are increased leading to a smoother (and usually more informative, flexible and robust) signal.

Sunspot numbers: a quantity that measures the number of temporary dark spots in the sun surface, where zero reflects no dark spots observed on a certain date.

Time series: data points indexed in time order

## Introduction

Strokes are the second leading cause of death and disability worldwide ( Mozaffarian *et al.,* 2015). Strokes share all of the recommended interventions for chronic noncommunicable diseases (NCD): life-style modifications, including a low fat/salt/sugar diet, moderate physical activity, discontinuation of smoking, sufficient sleep, and control of arterial blood pressure, and, if necessary, pharmacologic therapy ( Mozaffarian *et al.,* 2015). In addition, risk factors such as chronic stress, underlying diseases, such as obesity, diabetes mellitus, chronic obstructive pulmonary disease, and renal insufficiency, as well as predisposing genetic factors, have been implicated in stroke morbidity and mortality ( Malik & Dichgans, 2018). The incidence and prevalence of stroke subtypes vary greatly, depending on ethnicity and country income. As stroke statistics fail to cover all etiologies, some remain unknown.

Stroke has been previously associated with solar activity ( Halberg *et al.,* 2001; Otsuka *et al.,* 2001; Stoupel *et al.,* 1995; Stoupel *et al.,* 1996). Such activity, as indexed by sunspot numbers, has been generally associated with health ( Feigin *et al.,* 2014; Halberg *et al*., 1998; Petropoulos & Geronikolou, 2005). However, relevant chaos and trend analyses in local populations have been limited but strongly suggested ( Reinberg *et al.,* 2017).

Our aim was to investigate the dynamics and trends in the selected time series, to determine sunspot numbers vs. daily and monthly stroke deaths in synchronized periodicities with gradual time delays (chronomes), and to define a sunspot number threshold beyond which stroke death events may be influenced.

## Methods

In this study we focused on monthly stroke mortality events between 1985 and 1989, based on the underlined cause of death data (ICD-9 Table 5.3: recode 430, Table 5.4: recode 200) from the archives of Piraeus Civil Registry ( Geronikolou & Zikos, 1991).

The sunspot numbers were derived from the archives of measurements published by *Solar Geographical Data*. Sunspot Number, denoted Rz (Zurich number), is defined as: *Rz* = K(10g + f), where g is the number of sunspot groups visible on the Sun, f represents the total number of individual spots visible; and K is an instrumental factor to take into account differences between observers and observatories.

 The stroke death rate in Piraeus, was calculated using the formula (number of all deaths per year per 1000 people in June 30 ^th^, year x). The overall death rate was calculated with the denominators provided by the 1981 census ( Geronikolou & Zikos, 1991).

In the analysis of our short time series, we employed fast Fourier transform (FFT) analysis and the singular spectrum approach (SSA). Thus, we first performed a square root transformation of the sunspot time series. We subsequently analyzed the second time series of the stroke deaths using the SSA, to find the principal components that formulated it using principal component analysis (PCA). We applied Pearson correlation analysis to detect the coefficients of variation between the principal components of the sunspots and the strokes time series. All calculations were performed with MATLAB 7 software.

## Results

We focused on monthly stroke deaths, based on all death events archived in the local Civil Registry ( Geronikolou & Zikos, 1991). There were 792 stroke deaths out of 4324 total deaths events distributed in the 60 months of the quinquennium (1985–1989) examined. Over 54% were women and over 61% occurred at ages over 69 years. The stroke death rate (stroke deaths in year *x*/overall deaths in year *x* × 100) was calculated as 17.668 in 1985, 20.089 in 1986, 19.372 in 1987, 17.647 in 1988, and 15.531 in 1989. The overall death rate (all deaths in year *x*/local population in year *x* × 100) was 5.5 in 1985, 4.4 in 1986, 4.9 in 1987, 3.5 in 1988, and 3.7 in 1989.

**Figure 1.**
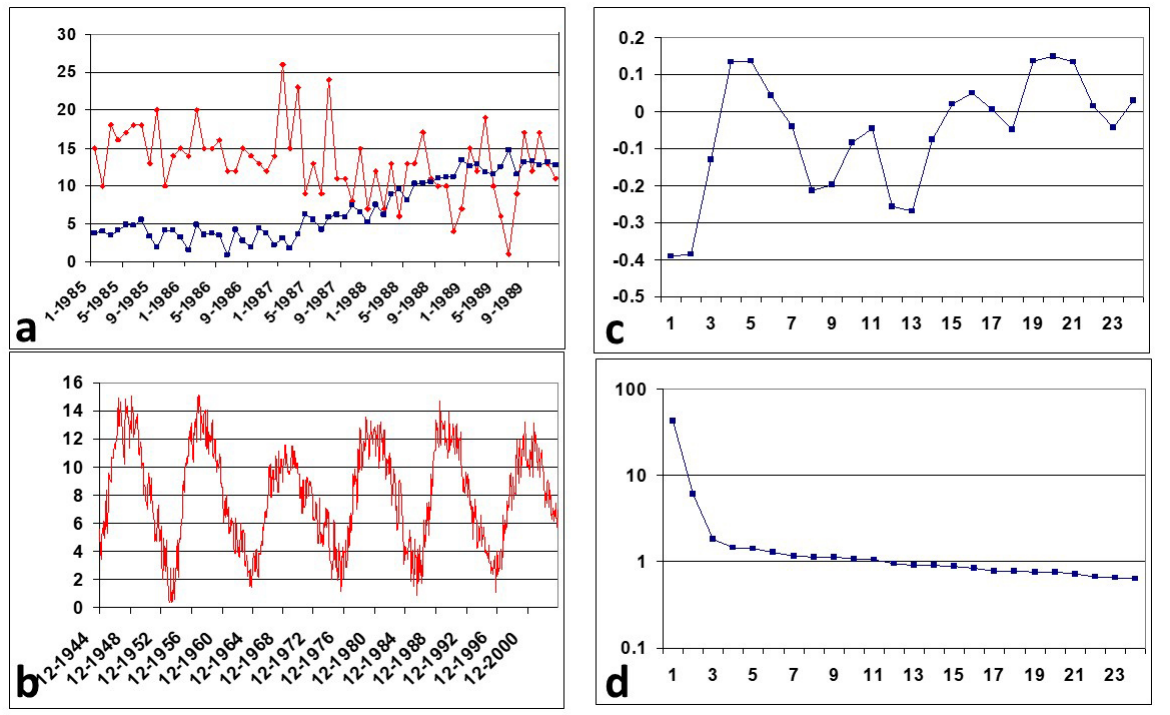
Singular spectrum analysis results. ( **a**) Time series of stroke deaths and sunspots from January 1985 to December 1989. A square root transformation is applied to the time series of the sunspots. ( **b**) Time series of sunspots from December 1984 to November 2004. ( **c**) Pearson correlation coefficient between the principal components of the sunspots and strokes time series. ( **d**) Singular values of the transformed sunspots time series. The noise plateau clearly begins at the third-ordered singular value.

The observed time series of both monthly and daily sunspot numbers and monthly and daily stroke death events in Piraeus between 1985 and 1989 are illustrated in Figure 1a. The PCA distinguished two principal components, as shown in Figure 1b: 6.8 and 20 days. The sunspot numbers observed (1944–2004) transformed to squared roots are described in Figure 1c. The singular values of the transformed sunspots time series showed that the noise plateau began at the 3 ^rd^ ordered singular value ( Figure 1d). Thus, monthly sunspot numbers by squared root variation and their violent fluctuation of over 35% was correlated to monthly stroke mortality, establishing a negative correlation between the two time-series (sunspot numbers and deaths by stroke) ( Figure 1b, d). FFT showed frequencies of 3.5 and 6.85 days.

Data on stroke deaths and sunspots by month are available as *Underlying data* ( Geronikolou & Leontitsis, 2020).

## Discussion

Mapping time-structures is a rapidly growing scientific field, enriching P4 medicine (predictive, preventative, personalized, participatory medicine) models with chronotherapy aspects ( Yan, 2015). Human biological clocks are intensively studied, as they represent adaptive body mechanisms necessary to assist with homeostatic changes caused by the environment, possibly including solar activity. These mechanisms have not been extensively investigated in Mediterranean populations. Sunspots have been framed for communicable (CDs) and non-communicable disorders (NCDs), same as epidemics ( Hrushesky *et al.*, 2011; Stoupel *et al.*, 2003; Stienen *et al.*, 2015; Yeung, 2006)

Chronic NCDs account for over 70% of early deaths worldwide, while stroke is the second leading cause of death and disability; the latter is associated with high expenses in health services, and constitutes a public health challenge ( WHO, 2018). This challenge is progressively increasing, considering the large population migrations that take place on the planet because of ethnic conflicts, economic crises and climate changes. Stroke has been associated with various risk factors, such as lifestyle-related eating habits, tobacco and/or alcohol use, and decreased physical activity, underlying comorbidities, such as obesity, hypertension, dyslipidemia, diabetes mellitus type 2, lung and kidney failures, etc., as well as exposure to environmental pollutants. Genetic propensities also contribute to various manifestations of the chronic noncommunicable diseases. Socioeconomic and geographic disparities have been suspected, while heliomagnetic influences have been proposed as possible etiologic contributors to human pathology ( Halberg *et al.*, 1998; Stienen *et al.*, 2015; Stoupel *et al.*, 1996; Stoupel *et al.*, 2003).

Mortality data meeting validity and credibility criteria are a *sine qua non* in the study of stroke incidence ( Feigin & Hoorn, 2004). Our study focused on stroke mortality in the largest Mediterranean port (Piraeus), ranked as the third most populated city in Greece. Moreover, its population is representative of the urban populations in Greece ( Geronikolou & Zikos 1991). The quinquennium 1985–1989 was chosen, because, until then, Greece had a rather robust diet (low fat/sugar, proteins and vegetables/fruits daily, pure olive oil almost exclusively) and genetic homogeneity, while environmental pollution was limited. In this period, these major confounding factors were thus not present: major pollution, nonstandard diet, foreign gene flow. The data used in this study were original and based on the reported underlined cause of death ( Geronikolou & Zikos, 1991). Importantly, the quinquennium selected was a period when the local population was of the same origin, while only small differences in the socioeconomically stratified levels were present. The covariates related to diet, hygiene and culture were stable in this period and, thus, they could be safely assumed as such.

The selected time period 1985–1989 emerged as an appropriate time to provide good reference observations, credible correlations, and future comparisons, and, hence, high inferential precision. Importantly, this period, although relatively short, included the maximum of the 22 ^nd^ solar cycle: July 1989 (maximum 157.6 or smoothed sunspot numbers 18.9), as well as the minimum of the 21 ^st^ solar cycle (minimum 13.4 or smoothed sunspot numbers 12.3). The 21 ^st^ solar cycle lasted 10.3 years, beginning in June 1976 and ending in September 1986. The 22 ^nd^ solar cycle lasted 9.7 years, beginning in September 1986 and ending in May 1996. Sunspot numbers correlation with other solar activity indices [UV/EUV, F10.7 flux (noontime measurement of the solar radio flux at a wavelength of 10.7 cm), Mg II] persisted at the same levels until 2000 ( Bruevich *et al.,* 2014; Floyd *et al.,* 2005). More importantly, they interact linearly except for the minima and maxima of the 11-year solar cycle, while these correlations vs sunspots and F10.7 flux, were shown to reach the lowest levels twice in each cycle ( Bruevich *et al.,* 2014). Furthermore, the same study showed that a double-peak structure was observed in cycle 22 but not in cycle 21. These phenomena enhance the validity of our results, as the examined period comprise the minimum of the 21 ^st^ and the beginning of the maximum of the 22 ^nd^ cycle. It is established that the sunspot minima coincide with an increased flux of cosmic rays, whereas, in sunspot maxima the heliosphere is shielded by planetary magnetospheres- a phenomenon known as “Forbush decrease” ( Raghav *et al.,* 2017). Thus, the latter merit future investigation in this population and similar ones. The sunspot numbers do not affect Earth directly; however, the solar wind emanating from solar activity affects stratospheric ozone layer density, whose ionization promotes health morbidity on inhabitants, including the prevalence of strokes ( Feigin *et al.,* 2014; Petropoulos & Geronikolou, 2005).

It has been suggested that chaos and trends in local evidence are lacking ( Halberg *et al*., 1998), and this study addresses this need. Chaotic dynamics analyses could unravel unknown patterns of stroke epidemiology -whose causes are not fully understood. Our work postulates that there is an inverse relation in two time series, between the timing of sunspot numbers and stroke deaths, a hypothesis posed by previous investigations ( Geronikolou & Leontitsis, 2005; Geronikolou & Petropoulos, 1996; Stoupel *et al.,* 1999). We showed that an over 35% change in the sunspot numbers, shifted the upwards trend of stroke deaths with a delay of two months.

The interaction of living organisms and their functions with solar radiation has been previously described ( Halberg *et al*., 1998; Reinberg *et al.,* 2017). This consisted mainly of protein secretion studies, and, less extensively of local population dynamics. The molecular interactions network approach, where the inter-species functional interactome of nuclear steroid receptors (R1) constructed on orthologues was employed ( Geronikolou *et al.,* 2018). R1 has interspecies dimensions and thus has evolutionary value extending from insects to humans, that is, from early life eras till now. Solar activity exposure was certainly omnipresent before life appeared on the planet. Similar cycles existed, such as those detected in our study, although the rotation of our planet around its axis and around the sun were faster than they are today ( Reinberg *et al.,* 2017). R1 includes genes and their products involved in circadian rhythms, while its major hub NCOR1 in macrophages blocks the pro-atherogenic functions of peroxisome proliferator-activated receptor gamma (PPARγ) ( Oppi *et al.,* 2020), greatly implicating stroke pathophysiology. PPARγ has a pleiotropic role in intracerebral hemorrhage ( Zhao *et al.,* 2015) and ischemic brain injury ( Culman *et al.,* 2007). It is likely that as soon as R1 is disrupted, the atherogenic and/or other pathological processes progress dramatically, with potentially lethal consequences. Here, both FFT and SSA revealed a novel common cycle of 6.8 days in Zurich numbers and stroke deaths. The cycle is smaller than the known anthropogenic circaseptan rhythm ( Reinberg *et al.,* 2017). The 17-ketosteroids were also found to have a <7-day cycle ( Hamburger *et al.,* 1985), confirming the steroid contribution to the phenomenon seen in R1 and herein.

It has been previously reported that the geomagnetic disturbance indices K _p_ and aa take the value of 6.75 ( Halberg *et al*., 1998), but without a clear association to the biota (living organisms -flora/fauna/humans). Our finding, apart from its novelty, provides a new insight in stroke epidemiology: the observed patterns suggest an endogenous natural rhythm of renewing populations.

Our work demonstrates a phase shift resulting from violent fluctuation in sunspots variation (>35%) with a clear correlation to monthly stroke deaths. A phase delay of two months was observed between the physical triggering and the death incidence shift. This should be investigated in the future over different and/or even longer periods of time and in different and/or larger populations. Still, the violent fluctuation of 35% of sunspots appears to be a hazard for mortality and, we assume, morbidity. Thus, future medical practice should probably take account of chronopathology ( Stienen *et al.,* 2015) so as to prevent stroke mortality shifts (chronotherapy and chronoprevention plans).

## Conclusions

Our work clearly established that sunspot numbers and stroke mortality were inversely correlated, and that a violent fluctuation of sunspot numbers over 35% shifted monthly mortality with a phase delay of two months. In addition, a common, novel, non-anthropogenic chronome of 6.8 days in solar activity (sunspot numbers) and stroke mortality was revealed. Time structure patterns evaluated with non-linear methods revealed new information on the stroke epidemic, and, thus, contributed to precision inference and the need of sophisticated public health policy planning.

## Data availability

Figshare: Singular Spectrum Analysis of monthly stroke deaths and mean monthly sunspot numbers. https://doi.org/10.6084/m9.figshare.12644981.v2 ( Geronikolou & Leontitsis, 2020).

This file contains the incidence of stroke deaths by month for 1985–1989 and the incidence of sunspots by month for 1944–2004.

Data are available under the terms of the Creative Commons Attribution 4.0 International license (CC-BY 4.0).
