# Supplementary material for: Cyclic stroke mortality variations follow sunspot patterns
Source: F1000Res. 2020 Dec 22;9:1088. Originally published 2020 Sep 3. [Version 2] doi: 10.12688/f1000research.24794.2 (PMC7667520; doi:10.12688/f1000research.24794.2)
Supplement: Peer review history

# Peer review: Reviewer response for version 1

**DOI:** 10.5256/f1000research.27352.r70999

**Published:** 2020-11-13

**Authors:** Kateřina Podolská

**Affiliations:** 1 Department of Ionosphere and Aeronomy, Institute of Atmospheric Physics, CAS, Prague, Czech Republic

In this manuscript, the authors propose non-anthropogenic 6.8 days cycle of stroke-related death events in Pireus port during 1985-1989 period.

 Reliable data sources of solar and magnetic activity are used.

 I suggest to answer the following questions and comments before the manuscript will be

 accepted for indexing:

 Please provide codes of underlined cause of death from the of the International Classification of Diseases (ICD-9) under which was analyzed stroke-related death events registered.

 Have you tried to use as another proxy of the solar activity in your analysis also Solar radio flux F10.7? The Solar radio flux reflects the nature of the process in a better way.

 The discussion part should be enlarged with consideration different dynamic of geomagnetic and solar activity during the ascending and descending phase of the solar cycle. Influence of this effect in changes of mortality from cardiovascular diseases was publicized.

 The effect of cosmic rays during the the solar cycle minima, to which human physiology is sensitive, should be also mentioned.

Is the work clearly and accurately presented and does it cite the current literature?

Yes

If applicable, is the statistical analysis and its interpretation appropriate?

Yes

Are all the source data underlying the results available to ensure full reproducibility?

Yes

Is the study design appropriate and is the work technically sound?

Yes

Are the conclusions drawn adequately supported by the results?

Yes

Are sufficient details of methods and analysis provided to allow replication by others?

Yes

Reviewer Expertise:

Stochastic Modeling, Demographic Analysis, Solar Terrestrial Interactions, Solar Flares

I confirm that I have read this submission and believe that I have an appropriate level of expertise to confirm that it is of an acceptable scientific standard.

# Peer review: Reviewer response for version 1

**DOI:** 10.5256/f1000research.27352.r71839

**Published:** 2020-11-03

**Authors:** Emmanuel Poulidakis

**Affiliations:** 1 Department of Cardiology, Evagelismos General Hospital, Athens, Greece; 2 Hôpital Privé Saint-Martin , Caen, France

Overall it is a good work, in a rather unusual subject, but seems to well support its arguments. 

 However, I believe that while its point is interesting, it does not sufficiently explain many of the terms used, as if addressing only those interested in the same field of research, somehow barring less expert audience. I suggest that in many cases, a few more phrases could make a difference. 

 For example, explaining certain terms (e.g. Sunspot numbers and time series) could make the article readable by a less specialized audience. I had to conduct research on my own to better understand these concepts. I would also suggest expanding the paragraph at the end of the introduction, detailing the aims of the study. I have attached a pdf copy with highlighted sections about possible corrections.

 Another observation is the structure of the abstract, which in my opinion should be divided into sections (background/methods/results/conclusion). 

 I noted a few spelling errors, highlighted in the pdf file.

Is the work clearly and accurately presented and does it cite the current literature?

Yes

If applicable, is the statistical analysis and its interpretation appropriate?

I cannot comment. A qualified statistician is required.

Are all the source data underlying the results available to ensure full reproducibility?

Yes

Is the study design appropriate and is the work technically sound?

Yes

Are the conclusions drawn adequately supported by the results?

Yes

Are sufficient details of methods and analysis provided to allow replication by others?

Yes

Reviewer Expertise:

Heart failure, echocardiography, interventional cardiology.

I confirm that I have read this submission and believe that I have an appropriate level of expertise to confirm that it is of an acceptable scientific standard.
